# A randomized controlled trial of an Internet-based self-help skill strengthening (ISSS) intervention for secondary school teachers: a transdiagnostic intervention for common mental health problems

**DOI:** 10.1186/s12888-026-08063-4

**Published:** 2026-04-17

**Authors:** Xue Yang, Phoenix K. H. Mo, Joseph T. F. Lau, Stanley S. M. Ho, Winnie W. S. Mak

**Affiliations:** 1 The Jockey Club School of Public Health and Primary Care, Faculty of Medicine, The Chinese University of Hong Kong, Shatin, New Territories, Hong Kong; 2 CUHK Shenzhen Research Institute, The Chinese University of Hong Kong, Shen Zhen, China; 3 Faculty of Education, The Chinese University of Hong Kong, Shatin, New Territories, Hong Kong; 4 Department of Psychology, The Chinese University of Hong Kong, Shatin, New Territories, Hong Kong

**Keywords:** Randomized controlled trial, Internet-based, Problem-solving therapy, Secondary school teachers, Common mental health problems

## Abstract

**Background:**

Mental health is crucial for educators, as it impacts their ability to effectively teach and engage with students. This two-arm parallel group randomized controlled trial (RCT) aims to investigate the efficacy of a transdiagnostic and low-intensity Internet-based self-help skill strengthening (ISSS) selective intervention based on problem-solving therapy (PST) for secondary school teachers with common mental health problems.

**Method:**

Participants included secondary school teachers with probable depression/anxiety in Hong Kong. Participants were evenly randomized into the intervention and control groups based on computer-generated random number and block randomization (size of six). The primary outcomes were depressive symptoms and anxiety symptoms. Secondary outcomes included perceived stress, self-efficacy, work performance, and psychological well-being. We also tested problem solving and time management as potential mediators.

**Results:**

There were 179 participants who finished the project (ISSS versus control = 94 versus 85). Linear mixed model showed significant main effect in time, *F* [2] = 11.29, *p*<.001, group, *F* [1] = 7.93, *p*=.007, and time by group interaction (*F*(2, 510.04) = 4.41, *p*=.013) on depressive symptoms, showing that intervention group had a greater improvement on level of depressive symptoms than control group over time. Similar results were found for anxiety symptoms, with significant main effect in time, *F* [2] = 13.81, *p*<.001, group, *F* [1] = 4.77, *p*=.032, and time by group interaction, *F*(2, 508.04) = 3.33, *p*=.037. Time management significantly and fully mediated the effects of groups on depressive symptoms at 6-month follow-up.

**Discussion:**

It suggests that ISSS based on PST may be an effective transdiagnostic intervention to reduce depression and anxiety among secondary school teachers in Hong Kong. The identified underlying mechanisms can help to improve and maintain the efficacy of online PST.

**Trial registration:**

The RCT protocol was pre-registered at the ClinicalTrials.gov registry (NCT04564014) on September 22, 2020 prior to data collection.

**Supplementary Information:**

The online version contains supplementary material available at 10.1186/s12888-026-08063-4.

## Introduction

Teaching is attentionally, socially, and emotionally demanding. It is consistently ranked as one of the top jobs in terms of stress-related health problems [1, 2]. Many stress factors contribute to teachers’ stress and emotional problems, such as administrative tasks, teaching-related tasks (e.g., conducting classes), and student-related issues (e.g., students’ problematic behaviors) [3–5]. A meta-analysis study demonstrated that the prevalence of high levels of burnout among secondary school teachers was significant worldwide [6]. Ma et al.’s meta-analysis reported that the prevalence of stress among teachers was 62.6%, anxiety was 36.3%, and depression was 59.9% [7]. Another meta-analysis revealed that the prevalence of burnout varied between 25.12% and 74%, anxiety symptoms between 38% and 41.2%, and depression among teachers from 4% to 77% [3]. Hong Kong (HK) secondary school teachers often experience high stress due to the excessive workload and administrative burdens, high-stakes examination culture (HK Diploma of Secondary Education Examination), diverse and demanding student needs, and high parental expectation. A recent report stated that 91.2% of 1240 HK teachers perceived their work-related stress as excessive or somewhat excessive [5], and Chong and Chan [8] revealed that 75.8% of teachers experienced anxiety and 54.7% had depression among a sample of 1710 HK teachers. Effective interventions for alleviating teachers’ common mental health problems and enhancing skills to cope with work-related stress are warranted. However, such evidence-based service is lacking. To our knowledge, only 5 randomized controlled trial (RCTs) have been conducted overseas to address common mental health problems among secondary school teachers [9–13]. One of them was Internet-based Problem-Solving Training (iPST) and reported significant reductions in depressive symptoms (*n* = 150) [9]. Internet-based stress management interventions (iSMIs) demonstrated effectiveness in reducing depression and anxiety among teachers (*n* = 200) [13]. The other three were mindfulness-based interventions and found significant effects on job stress or anxiety (*n* = 58 to 191). No related RCT on common mental health problems in secondary school teachers was found in HK.

Problem-solving training (PST) is a transdiagnostic low-intensity intervention for common mental problems and is recommended by the World Health Organization (WHO). It focuses on training in adaptive problem-solving attitudes and skills [14], based on the relational/problem-solving model of stress and well-being that assumes problem solving plays an important role as a mediator/moderator in the relationship between stressful problems and well-being. Meta-analysis studies have demonstrated that PST is as effective as other psychosocial therapies and medication treatments and is more effective than no treatment and support/attention control groups [15]. PST is also recommended by stepped-care models as a minimal treatment for people with less severe depression/anxiety [16]. Previous studies have demonstrated its effectiveness in treating patients with depression/anxiety [17, 18]. Globally, we only identified one PST study among teachers [9], while others mainly used mindfulness-based approach. Compared with mindfulness-based therapy and full cognitive-behavioral therapy (CBT), PST is a brief, low-intensity, little time/resource-consuming, and cost-effective intervention [19]. Furthermore, PST does not aim to cognitively restructure teachers’ stressful feelings, but to help solve the source of their strain by enhancing their ability to analyze problems, identify problem severity, and assess the impact of alternative solutions. It is solution-oriented and empowers individuals to take an active role in finding solutions to problems and stress; thus, perceived stress would decrease and psychological well-being and self-efficacy in coping with problems and stressful situations would increase when teachers strengthen their problem-solving skills [9].Since secondary school teachers encounter many work-related challenges (e.g., interpersonal conflicts with students/students’ parents/colleagues), a skill strengthening program to enhance work-related problem-solving skills would be particularly effective in reducing teachers’ emotional problems and stress. In addition, one of the significant work-related problems of teachers is that they often feel it impossible to fit all tasks (e.g., the long-term goals of the classroom, the immediate educational needs of the students, and the large volume of paperwork that comes with every assignment) into the allotted time frame. To help teachers cope with this problem, time management training is also warranted to enhance their effective use of time while performing certain goal-directed activities. Three components of the time management training include setting goals and priorities, mechanics (i.e., making lists and scheduling), and preference for organization [20]. Such skills would help teachers to meet their job demands, reduce their job stress and anxiety, and improve their performance and emotional health [21].

Internet-based interventions have a number of advantages, such as being anonymous, sustainable, easily accessible, convenient, flexible, low-cost, interactive, and tailored to the individual. Delivering health interventions via the Internet will especially benefit teachers, as they often face great time and job pressure and highly hesitate to disclose their mental problems to seek help due to their job nature. Although most previous studies did not focus on teachers, the CBT-based interventions indicated that self-help internet interventions could reduce depression or anxiety across different population groups [22].

### The present study

The current project developed an Internet-based guided self-help intervention among secondary school teachers based on PST (the Internet-based self-help skill strengthening-the ISSS programme). The intervention aimed to reduce depression/anxiety symptoms through strengthening skills to cope with work-related problems; it included problem-solving and time management trainings. The primary objective of this RCT study was to assess the efficacy of such an intervention in reducing depression/anxiety symptoms, compared with a control group. Primary outcomes were depression/anxiety symptoms, and secondary outcomes included perceived stress, self-efficacy in coping with problems, work performance, and psychological well-being. Furthermore, we examined potential mechanisms (mediators) that may explain the efficacy, including problem solving and time management behaviors. It is hypothesized that the ISSS intervention would be more effective in reducing depression and anxiety and enhancing self-efficacy in coping with problems, work performance, and psychological well-being than the control. In addition, the ISSS would increase problem solving and time management behaviors, which would in turn improve the primary and secondary outcomes.

## Methods

This was a parallel two-arm RCT, which was conducted and reported in accordance with the CONSORT guidelines. Inclusion criteria included (1) being a secondary school teacher in Hong Kong, (2) speaking Chinese, (3) having access to the Internet, and (4) having probable depression or probable anxiety. These reflect the formal study protocol and correct a summary inconsistency in the trial registration. Consistent with the protocol, those with high suicidal risk were excluded. Contents of the intervention were designed by a research team, which included health psychologists, a clinical psychologist, an epidemiologist, and a former secondary school principal. Techers from international schools were excluded because their working experience and school resources may significantly differ from the local school teachers. The RCT protocol was pre-registered at the ClinicalTrials.gov registry (NCT04564014) on September 22, 2020 prior to data collection. Evaluations were assessed at baseline (T0), one month after the intervention (T1), and six months after the intervention (T2). The RCT was conducted during January 2022 to June 2024.

### Measures

All the measures were validated scales with good reliability in the current sample (Table 1). Demographic information (e.g., age, gender, education, marital status, school types) was collected at baseline. The use of other mental health services and stressful life events during the program were administered at follow-ups.

**Table 1:** Cronbach’s alpha of each scale

	Baseline	Post	Follow up
PHQ	0.67	0.75	0.77
GAD	0.84	0.86	0.95
GSE	0.90	0.89	0.91
PSQ	0.84	0.74	0.85
JP	0.93	0.91	0.89
WHO-5	0.92	0.85	0.87
PSI	0.86	0.74	0.72
TMBS	0.91	0.85	0.81

Note. PHQ = depression; GAD = anxiety; GSE = self-efficacy; PSQ = perceived stress; JP = job performance; WHO-5 = well-being; PSI = problem-solving; TMBS = time management

#### Screening and primary outcome measures (Appendix 1 for details of the measures)

The Patient Health Questionnaire (PHQ-9) [23] and Generalized Anxiety Disorder Scale (GAD-7) [24] were used to assess depression symptoms and anxiety symptoms, respectively. The cutoff for probable depression or probable anxiety (mild to severe) was 5 [25]. The Chinese version of both scales was reliable and valid for screening probable depression and probable anxiety [26, 27].

#### Secondary outcome and mediator measures

Secondary outcomes (perceived stress, self-efficacy, job performance, and well-being) and mediators (problem-solving and time management) were tested by the 20-item Perceived Stress Questionnaire (PSQ) [28, 29], the 10-item General Self-Efficacy Scale (GSE) [30], the 11-item item Performance Maintenance Scale (PMS) [31], the 5-item World Health Organization Well-Being Index (WHO-5) [32], the 35-item Problem Solving Inventory (PSI) [33], and the 33-item Time Management Behavior Scale (TMBS) [34], respectively.

### Data collection and procedure

Invitation letters and posters that included the significance and logistics of the study and contact number for inquiry were sent to all local secondary schools, teachers’ associations, and mental health associations. In addition, advertisements and announcements were posted on popular online networking platforms (e.g., Instagram, Facebook). Interested participants contacted our research assistants by phone. Research assistants described details of the study including the aims, length, logistics, involvement of the participants, and randomization of participants to conditions. Verbal informed consent was sought, and the study applied a double-blind design. Eligible individuals were invited to finish a baseline questionnaire that took 15 min. They were then evenly randomized into the intervention and control groups (1:1), based on computer-generated random number and block randomization (size of six). The sequence was generated by an independent statistician using R statistical software (version 4.2.1) with the ‘randomizeR’ package. Due to the nature of the behavioral intervention, participants could not be blinded to group allocation. However, investigators and outcome assessors were blinded to the randomization status of participants throughout the study period.

An assistant assigned each participant a next sequential, unique study identification number and sent pre-written email to each participant. For those assigned to the intervention, this email contained education materials, their login instructions, and the first module. For those in the control group, the email contained education materials, confirmed their placement on the waitlist, and provided the date they would gain access. The principal investigators and research staff responsible for recruitment and outreach had no access to this part of the database.

### Intervention group

In addition to receiving education materials via email each week as the control group did, the intervention group also received the ISSS intervention. ISSS aimed to help participants acquire problem-solving (e.g., interpersonal problems with other teachers, students, and parents) and time management based on the treatment manual of the therapy [20, 35]. The essential principles are to enhance adaptive problem-solving attitudes (e.g., viewing problems as challenges) and skills (e.g., systematic problem-solving steps), and to improve time management behaviors (e.g., prioritization, scheduling).

#### Content and structure

Video introductions for each session and example teacher characters depicted the targeted problems and demonstrated implementation of coping/management techniques in a variety of situations. ISSS used metaphors, daily examples, and lively narrative stories to enhance motivation for participation and understanding of the intervention contents. Each module included a video introduction (approx. 10–15 min) featuring a health psychologist explaining the core concepts, animated narrative stories featuring example teacher characters (e.g., “Mr. Chan” struggled with disruptive students, “Ms. Yeung” overwhelmed by paperwork) to model the application of skills, and interactive exercises (e.g., In Session 2, a drag-and-drop exercise required users to sequence the correct problem-solving steps). Session 2–5 assigned exercise and homework to practice the learnt skills (e.g., a downloadable “Problem-Solving Worksheet” to apply the 6-step process to a real-life problem; a “Time Log” to track activities for a week). Within 48 h, participants would receive personalized written feedback (e.g., affirmation, clarification, encouragement, and suggestions) from an e-Coach on the exercises they had completed. For example, on a submitted Problem-Solving Worksheet, the e-Coach would praise the participant’s effort (“Well done on listing so many potential solutions”). Participants could contact e-Coach via the online system if they had any questions regarding the contents. Questions that were beyond the contents would be referred to the health psychologist (the corresponding author) by e-Coach. The session topics and its subsection contents are listed in Table 2.

**Table 2:** Structure and Content of the Internet-based Self-help Skill Strengthening (ISSS) Intervention

Session & Topics	Components	Key Skills & Learning Objectives	
Session 1 Introduction	-Information about symptoms, prevalence, causes, consequences, and treatment of emotional problems -Overview of problem solving skills and time management	To (a) highlight the importance of emotional problems and (b) adaptive coping, and (c) enhance their participation motivation	
Session 2 Problem solving skills	-Basic principles of problem-solving training and exercises	To enhance skills including (a) describing the problem, (b) listing possible solutions, (c) choosing the best solution, (d) making a plan for carrying out the solution, (e) actually carrying out the solution, (f) evaluating the success.	
Session 3 Interpersonal problem solving	-Video introductions and examples of applying problem-solving skills to cope with interpersonal problems with students/students’ parents/colleagues	To apply problem-solving skills to cope with interpersonal problems and manage interpersonal conflict.	
Session 4 Time management skills	-Basic principles of time management training	To enhance skills including (a) setting goals, (b) prioritizing, (c) making lists, (d) scheduling and planning, (e) organizing desk and papers, (f) dealing with procrastination, and (g) dealing with interruptions.	
Session 5 Maintenance and applying skills into general problems	-Review the learned skills, homework, feedback from e-Coach -Lecture on self-efficacy of keep using learned skills in both work and daily life	To help participants be able to use the learned skills to cope with problems occurring outside of therapy.	

#### Delivery platform

Each participant created a login ID and password for the Web-based programme participation. ISSS consisted of 5 sessions. Each session lasted about 60 min. Participants were advised to conduct one session per week and practice the learned techniques between sessions. If they did not finish the session, they could continue to finish it within the week and would receive reminders before finishing it. Participants also received reminders between sessions. The next session would be automatically available for the participant once he/she finished the previous session. Automated reminders would be sent to the participants if they had not logged into the website for over one week. Those failing to log-in after four reminders for the same session and not responding to email/messages were considered as dropout cases.

#### e-Coach support and training

The e-Coach was a research assistant with master’s degree in psychology and training in counseling. The e-Coach was supervised by health psychologists (the corresponding author and the co-author) and followed feedback guidelines according to a standardized manual, which was conceptualized based on a theoretical model for providing guidance in eHealth interventions and aimed to improve adherence to the web-based intervention [36]. In 3-day (24-hour total) sessions conducted by health psychologists, the e-Coach received training in concepts about emotional problems, problem solving and time management theories and techniques, by means of role-play, supervised practice, feedback, questions, and answers. The corresponding author met regularly with the e-Coach; continual and timely support was available to her throughout the intervention period.

#### Intervention fidelity

All content was scripted and pre-programmed into the web platform. The e-Coach followed a strict feedback protocol to ensure consistency. Furthermore, the supervising health psychologist (the corresponding author) conducted weekly meetings with the e-Coach and reviewed a random 20% of all feedback messages to ensure adherence to the protocol and address any questions.

### The control group

The control group received education materials about the following issues: [1] introduction to mental health and mental illness; [2] signs and symptoms of depression; [3] treatment of probable depression and available community resources; [4] signs and symptoms of anxiety; [5] treatment of probable anxiety and available community resources. The education materials were developed by health psychologists and a clinical psychologist of the research team. This is an active control group that corresponds to the condition described in our trial registration as a ‘wait-list control group’ that received informational materials. Participants would receive ISSS after completion of the follow-up evaluation.

### Ethical considerations

This study was in compliance with The Declaration of Helsinki and IGH-GCP to safeguard the interests of research participants and complied with research ethics principles. Informed concern was obtained from each participant. The clinical ethics approval was obtained from Joint CUHK-NTEC CREC (Ref#2021.130). To assess for potential harms, we reviewed all participant communications with the research team, examined reasons for dropout, and monitored open-ended feedback provided at post-test and follow-up assessments for any indications of negative effects related to the study procedures. As a result, there were no indications of adverse events or unintended harm related to the study.

### Data analysis

The study conducted in agreement with the CONSORT statement. Intention-to-treat (ITT) analysis was performed. Patterns of missingness were examined by comparing participants with complete data and those with missing data on key baseline demographic and outcome variables (e.g., age, gender, PHQ-9, GAD-7) using t-tests and chi-square tests. No significant differences were found (all *p* >.05), suggesting that the data were missing at random (MAR) rather than not missing at random (NMAR). Therefore, multiple imputation was conducted by using chained equations with R package (MICE) and all analyses were based on 100 datasets generated from multiple imputation to account for missing values. Independent t-tests and Chi-square tests were conducted to test between-group differences in baseline characteristics. Independent t-tests were used to describe the between-group differences in primary and secondary outcomes. While our pre-registered protocol specified an ANCOVA as the primary method, we elected to use Linear mixed model (LMM) to test the time and group effects on the primary and secondary outcomes in this report. This decision was made prior to examining the results and was based on the methodological advantages of LMM for our specific data structure. LMM is a more appropriate and flexible extension of the ANCOVA framework for longitudinal data as it: [1] accounts for the correlation between repeated measures within the same individual by including random effects; [2] robustly handles missing data under a missing-at-random assumption without the need for imputation, thereby using all available data; and [3] models the trajectory of the outcome over time. Models included fixed effects for time, group, and the time-by-group interaction, and a random intercept for participants to account for within-subject correlation. An unstructured covariance matrix was used. Attended sessions were controlled. Per-Protocol (PP) LMM was also conducted as a sensitive analysis. The potential mediation effects of problem solving and time management between intervention conditions and primary outcomes were tested by using path analysis and bootstrapping analysis. The analyses were conducted by IBM SPSS Version 27 and Amos. The significance level was set to 5%.

### Sample size calculations

Ebert’s iPST study reported a large effect size (Cohen’s d= 0.90) for the effectiveness of an Internet-based PST in reducing depression compared with the wait-list control among teachers [9]. Using G*Power 3.1, it is estimated that a total of 128 participants (64/group) are needed to detect a medium effect size (f=0.25), power=0.80 for LMM on the primary outcomes (depressive symptoms/anxiety symptoms).

## Results

### Descriptive statistics of the participants

**Fig. 1:**
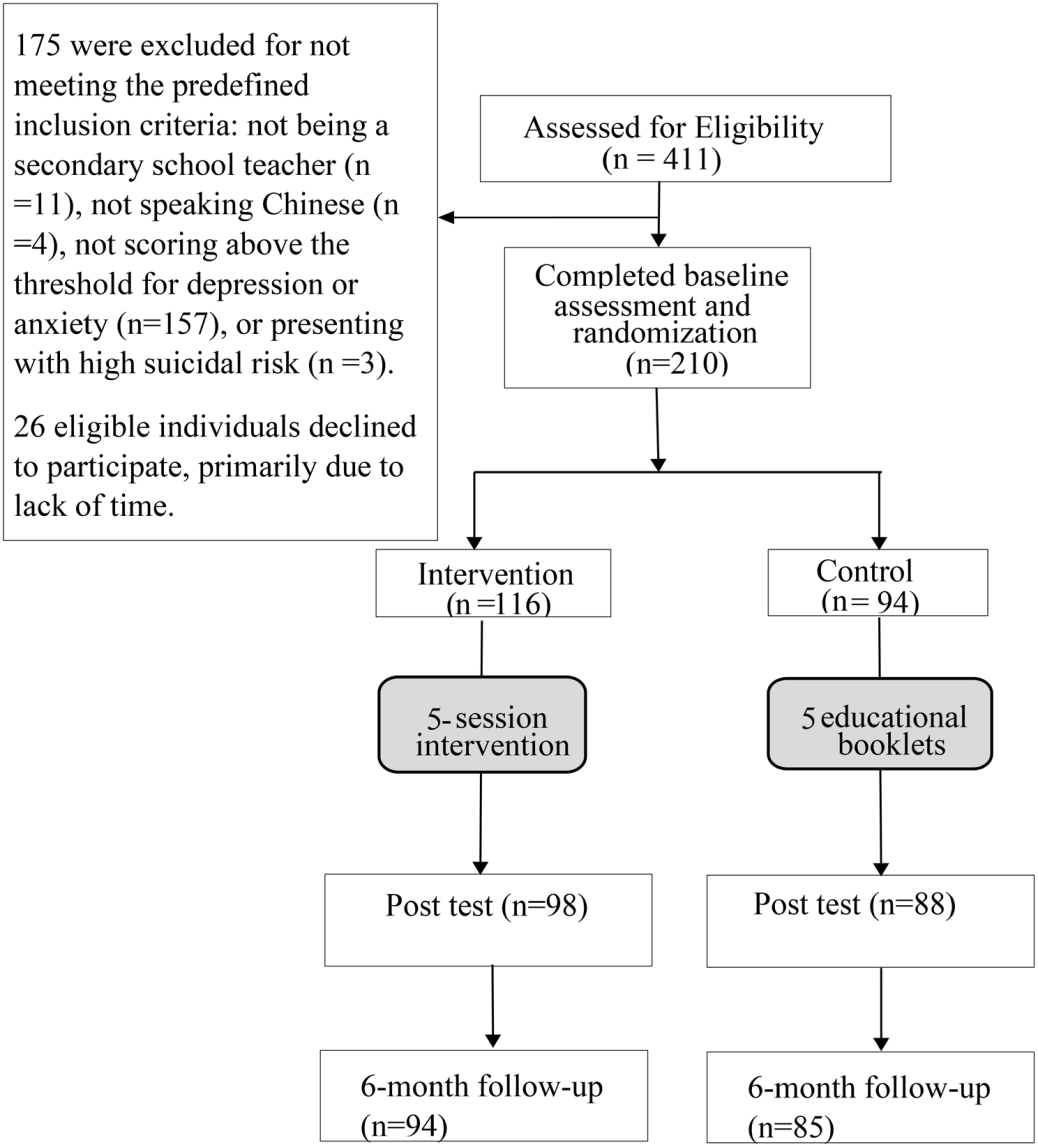
Flow chart of the study

Among 411 screened participants, 210 (51%) fulfilled the inclusion criteria, and 179 (dropout rate: 14.8%) finished at least 3 sessions of intervention (3 sessions: 82.4%, 4 sessions: 1.4%, 5 sessions: 16.2%) and evaluations (Fig. 1). In the control group (*n* = 85), 63.5% female, while in the ISSS intervention group (*n* = 94), 72.3% were female (Table 3). In the control group, 70 participants met the criteria for probable depression (PHQ score ≥ 5) and 71 for probable anxiety (GAD score ≥ 5) at baseline. In the intervention group, 81 participants had probable depression and 86 showed probable anxiety at baseline. At post-test, 56 participants were classified as having probable depression and 50 had probable anxiety in the control group, while in the intervention group, 48 participants had probable depression and 38 had probable anxiety. At the six-month follow-up, 60 participants had probable depression and 53 demonstrated probable anxiety in the control group, whereas in the intervention group, 59 had probable depression and 65 showed probable anxiety (Table 4). According to Table 5, the mean values of baseline self-efficacy (GSE_B) were significant between intervention group (25.51) and control group (23.44, *p*=.014). Significant differences were also found for depressive symptoms at post-test (PHQ_P) (intervention group vs. control group = 5.48 vs. 6.93, *p*=.029), anxiety symptoms at post-test (GAD_P) (intervention group vs. control group = 5.01 vs. 6.74, *p*=.017), well-being levels at post-test (WHO-5_P) (intervention group vs. control group = 17.09 vs. 15.46, *p*=.019), depressive symptoms at follow-up (PHQ_FU) (intervention group vs. control group = 6.40 vs. 7.72, *p*=.039), and time management levels at follow-up (TMBS_FU) (intervention group vs. control group = 120.80 vs. 117.29, *p*=.040).

**Table 3:** Background characteristics of the participants at baseline

Background variables	Control group (*n* = 85)	Intervention group (*n* = 94)	*P* value
Gender, n (%)			0.206
Male	31 (36.5%)	26 (27.7%)	
Female	54 (63.5%)	68 (72.3%)	
Age, Mean (SD)	40.06 (11.31)	41.53 (12.11)	0.405
Education, n (%)			0.442
Bachelor or below	34 (40.0%)	31 (33.0%)	
Master or above	51 (60.0%)	63 (67.0%)	
Marital status, n (%)			0.128
Never married	34 (40.0%)	34 (36.2%)	
Married/Cohabit	45 (52.9%)	55 (58.5%)	
Divorced/Separated/Widow	4(4.7%)	1 (1.1%)	
Others	2 (2.4%)	4 (4.3%)	
School type, n (%)			0.194
Aided school	64 (75.3%)	81 (86.2%)	
Government school	5 (5.9%)	3(3.2%)	
Private school	2 (2.4%)	0 (0%)	
Direct Subsidy Scheme School	14 (16.5%)	10(10.6%)	

Note. Chi-square tests were used for gender, education, marital status, and school type; an independent t-test was conducted for age

**Table 4:** Proportions of probable depression and probable anxiety

	Control group, *n* (%)		Intervention group, *n*(%)
Depression (Baseline)			
Mild (5–9)	42 (49.5%)		46 (48.9%)
Moderate (10–14)	26 (30.7%)		34 (36.1%)
Severe (> 14)	2 (2.4%)		1 (1.1%)
Anxiety (Baseline)			
Mild (5–9)	49 (57.7%)		50 (53.2%)
Moderate (10–14)	14 (16.5%)		27 (28.8%)
Severe (> 14)	8 (9.5%)		9 (9.7%)
Depression (Post)			
Mild (5–9)	38 (44.8%)		33 (35.1%)
Moderate (10–14)	16 (18.9%)		10 (10.6%)
Severe (> 14)	2 (2.4%)		5 (5.3%)
Anxiety (Post)			
Mild (5–9)	30 (35.2%)		24 (25.6%)
Moderate (10–14)	17 (20.0%)		10 (10.7%)
Severe (> 14)	3 (3.6%)		4 (4.3%)
Depression (Follow up)			
Mild (5–9)	29 (34.1%)		36 (38.3%)
Moderate (10–14)	20 (23.6%)		22 (23.4%)
Severe (> 14)	11 (13.1%)		1 (1.1%)
Anxiety (Follow up)			
Mild (5–9)	25 (29.4%)		36 (38.3%)
Moderate (10–14)	21 (24.7%)		22 (23.5%)
Severe (> 14)	7 (8.3%)		7 (7.6%)

Note. Depression was measured using the Patient Health Questionnaire-9 (PHQ-9), while anxiety was measured by the Generalized Anxiety Disorder-7 scale (GAD-7)

**Table 5:** Independent t-test for group comparisons in mean and standard deviation

	Control, Mean (SD)	Intervention, Mean (SD)	*p* value	Cohen’s d
PHQ_B	7.85 (3.52)	8.65 (3.43)	0.125	− 0.231
GAD_B	8.11 (3.95)	8.83 (3.84)	0.216	− 0.186
GSE_B	25.51 (5.50)	23.44 (5.68)	0.014	0.370
PSQ_B	30.72 (4.11)	31.64 (5.60)	0.269	− 0.186
JP_B	67.66 (15.50)	71.83 (17.92)	0.141	− 0.248
WHO-5_B	19.53 (4.49)	20.36 (4.63)	0.279	− 0.182
PSI_B	101.72 (10.87)	102.92 (16.75)	0.627	− 0.084
TMBS_B	111.72 (11.50)	115.77 (20.36)	0.151	− 0.242
PHQ_P	6.93 (3.52)	5.48 (4.62)	0.029	0.350
GAD_P	6.74 (4.55)	5.01 (4.51)	0.017	0.383
GSE_P	24.53 (5.68)	24.18 (4.93)	0.678	0.066
PSQ_P	29.72 (4.62)	30.48 (5.41)	0.229	− 0.149
JP_P	65.69 (15.93)	68.75 (14.18)	0.203	− 0.204
WHO-5_P	15.46 (4.14)	17.09 (4.52)	0.019	− 0.376
PSI_P	96.43 (11.99)	99.40 (10.58)	0.104	− 0.263
TMBS_P	116.86 (10.67)	117.19 (15.49)	0.880	− 0.024
PHQ_FU	7.72 (4.77)	6.40 (3.67)	0.039	0.311
GAD_FU	6.94 (5.38)	7.18 (4.80)	0.753	− 0.047
GSE_FU	26.44 (5.39)	25.30 (5.82)	0.182	0.201
PSQ_FU	29.72 (6.23)	29.54 (6.08)	0.203	0.192
JP_FU	66.57 (15.19)	64.94 (12.72)	0.438	0.117
WHO-5_FU	15.86 (5.08)	15.31 (4.27)	0.438	0.117
PSI_FU	95.60 (10.77)	96.32 (9.17)	0.636	− 0.072
TMBS_FU	117.29 (9.81)	120.80 (12.39)	0.040	− 0.312

Note. _B = baseline; _P = post-test; _FU = 6-month follow-up; PHQ = depression; GAD = anxiety; GSE = self-efficacy; PSQ = perceived stress; JP = job performance; WHO-5 = well-being; PSI = problem-solving; TMBS = time management

### Linear mixed models

Regarding depressive symptoms, results indicated significant main effect in time, *F* [2] = 11.29, *p*<.001, and in group, *F* [1] = 7.93, *p*=.007. The results also indicated significant time by group interaction, *F*(2, 510.04) = 4.41, *p*=.013, showing that intervention group had a greater improvement on level of depressive symptoms than control group over time (Table 6).

**Table 6:** Linear mixed models

	Control Mean (95%CI)	Intervention Mean (95%CI)	Group (F, *p* value)	Time (F, *p* value)	Group * time (F, *p* value)
PHQ	8.73 (7.42, 10.04)	7.20 (6.62, 7.78)	7.93, 0.007	11.29, < 0.001	4.41, 0.013
GAD	8.83 (7.33, 10.32)	7.46 (6.80, 8.13)	4.77, 0.032	13.81, < 0.001	3.33, 0.037
GSE	25.49 (24.79, 26.18)	24.31 (23.65, 24.96)	5.91, 0.015	4.06, 0.018	1.03, 0.358
PSQ	29.38 (28.67, 30.10)	29.89 (29.21, 30.56)	0.00, 0.317	1.97, 0.105	1.26, 0.139
JP	69.29 (63.17, 75.40)	69.31 (66.98, 71.64)	0.00, 0.991	2.82, 0.061	1.74, 0.176
WHO-5	18.30 (16.75, 19.86)	18.00 (17.30, 18.70)	0.23, 0.633	41.30, < 0.001	2.60, 0.075
PSI	100.67 (96.50, 104.85)	100.39 (98.56, 102.23)	0.03, 0.862	11.56, < 0.001	0.43, 0.654
TMBS	107.87 (103.12, 112.62)	115.66 (113.51, 117.82)	15.34, < 0.001	6.27, 0.002	0.87, 0.420

Note. PHQ = depression; GAD = anxiety; GSE = self-efficacy; PSQ = perceived stress; JP = job performance; WHO-5 = well-being; PSI = problem-solving; TMBS = time management

Similar results were found for anxiety symptoms, with significant main effect in time, *F* [2] = 13.81, *p*<.001, group, *F* [1] = 4.77, *p*=.032, and time by group interaction, *F*(2, 508.04) = 3.33, *p*=.037, showing that intervention group had a greater improvement on level of anxiety symptoms than control group over time.

Significant main effects in time were observed for self-efficacy (*F* [2] = 4.06, *p* = .018), well-being (*F* [2] = 41.30, *p* < .001), problem-solving (*F* [2] = 11.56, *p* < .001), and time management (*F* [2] = 6.27, *p* = .002). Only self-efficacy (*F* [1] = 5.91, *p* = .015) and time management (*F* [1] = 15.34, *p* < .001) showed significant group effects. No significant time by group interaction was found for these outcomes.

Results of PP LMM are presented in Appendix 1. Depressive symptoms showed significant time effects (*F* [2] = 5.91, *p* = .003) and group effects (*F* [1] = 4.97, *p*=.027), and the marginally significant time-by-group interaction was found (*F*(2, 298) = = 2.35, *p* = .097). For anxiety symptoms, significant main effects in time (*F* [2] = 7.37, *p*<.001), group (*F* [1] = 3.95, *p*=.048), and time by group interaction (*F*(2, 296) = 3.51, *p*=.031) were observed. For secondary outcomes, significant main effects in time were found in well-being (*F* [2] = 9.18, *p* < .001) and problem-solving (*F* [2] = 8.52, *p* < .001), while significant main effects in group was only found in time management (*F* [1] = 21.77, *p* < .001). The significant time by group interaction was found for self-efficacy (*F*(2, 298) = 3.17, *p*=.043).

### Path analysis

**Fig. 2:**
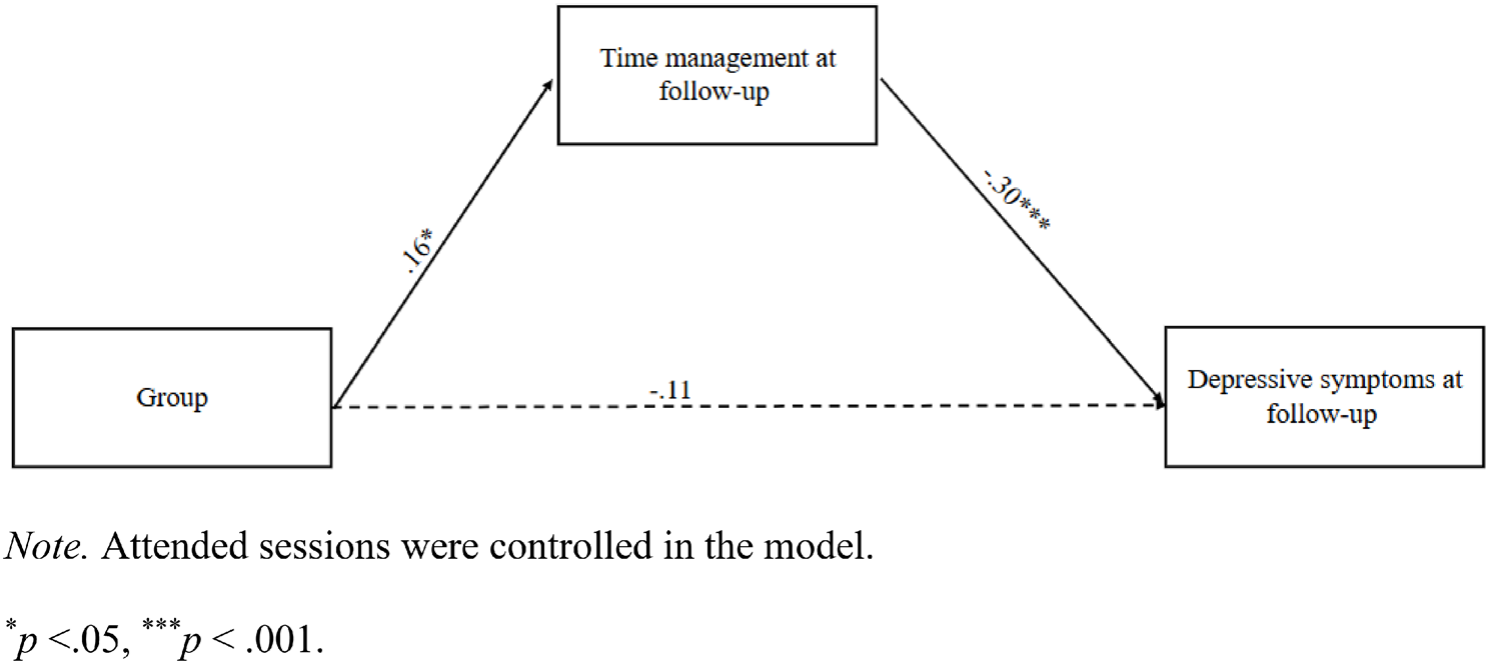
The mediating effects of time management at follow-up between intervention conditions and depressive symptoms at follow-up. Note. Attended sessions were controlled in the model. *p <.05, ***p <.001

Regarding the potential mediators/mechanisms (problem-solving and time management at post-test and follow-up), we only found the mediation model of time management at follow-up showed a good model fit (chi-square/df = 2.240, CFI=0.994, NNFI=0.910, RMSEA=0.080), and time management at follow-up significantly mediated the effects of groups on depressive symptoms at follow-up (*β*=-0.05, 95%CI [-0.10, − 0.02], *p*=.004). Group was significantly associated with time management at follow-up (*β* = 0.16, *p*=.035), and time management at follow-up was significantly associated with depressive symptoms at follow-up (*β*=-0.30, *p*<.001). The direct effect of groups on depressive symptoms at follow-up was not significant (*β*=-0.11, *p*=.133), suggesting a full mediation effect of time management (Fig. 2).

## Discussion

The present RCT study provides robust evidence on the efficacy of the ISSS for common mental health problems of secondary school teachers in HK. Specifically, our results indicated that the ISSS had long-term benefits for depressive symptoms and anxiety symptoms. The findings match those observed in the previous research on iPST [9], which demonstrated a greater reduction in depressive symptoms among German teachers. Beyond its efficacy for depressive symptoms, our study provides evidence that the ISSS intervention was also effective in reducing anxiety symptoms at post-test. This suggests that internet-based PST may function as a transdiagnostic intervention for common mental health problems. This is theoretically coherent because PST does not target disorder-specific symptoms (e.g., restlessness for anxiety, anhedonia for depression) but rather the underlying maladaptive coping processes that are common across both disorders, such as feelings of helplessness, avoidance, and poor confidence in managing life stressors. For teachers, whose stress is often multifaceted and does not fit neatly into a single diagnostic category, a transdiagnostic approach that enhances general problem-solving capacity may be more appropriate and scalable than disorder-specific protocols. Our results align with a growing body of literature supporting transdiagnostic interventions, particularly those based on CBT principles [37], but extend it by highlighting the specific transdiagnostic potential of a lower-intensity, problem-solving approach. This indicates that ISSS could serve as a versatile first-line intervention in a stepped-care model within the school setting, capable of addressing a range of emotional difficulties. Furthermore, self-efficacy showed significant time differences and group differences, which is consistent with the iPST study [9]. This efficacy may be due to the skill training for teachers to cope with the emotion-, work-, students-, and parents- related problems and perceived coping resources during ISSS. Such skills have been found to effectively promote their confidence in the ability to exert control over their own motivation, behavior, and social environment, and to facilitate healthy behavioral changes [38].

Time management had significant time differences and group differences. Time management at six-month follow-up was also a significant mediator to explain the maintained effectiveness of ISSS on depressive symptoms at six-month follow-up. It is interesting to find that time management was not a significant mediator at post-test but was significant at six-month follow-up. This delayed mediation effect is particularly intriguing when considered alongside the program’s completion rate. Although only 15% of participants attended the dedicated time management session (Session 4), we still observed a significant group effect on this skill. This suggests that the core mechanisms for improving time management were not isolated to a single session but were likely seeded earlier. It is possible that the foundational skills from Sessions 1–3 (e.g., overview of time management in session 1, goal setting in problem solving sessions) provided a foundation for participants to begin improving their time management. This delayed effect may occur because time management requires sustained practice to become an automatic habit. Participants needed time to consistently apply the learned skills and experience their benefits. Once proficiency was achieved through ongoing practice, the significant impact on reducing depressive symptoms emerged. This also explains why the behavioral skills training group sustained long-term gains, unlike the control group that received only educational information. To optimize future interventions, research must explore not just if an intervention works, but how it is used in daily life. A key question is how the frequency and quality of skill practice after the formal program ends contribute to sustaining gains. Understanding this naturalistic practice pattern can reveal the mechanisms of long-term change and guide the personalization of booster sessions or support tools for those who stop practicing.

A key strength of this study was the use of an active control condition rather than a passive waitlist. While waitlist controls are common, they cannot rule out the possibility that intervention effects are due to non-specific factors such as the mere expectation of receiving help, additional attention from researchers, or the simple passage of time. Our control group received structured psychoeducational materials, controlling for these factors. Therefore, compared with Ebert’s iPST study [9], the significant improvements in the ISSS group relative to this active control provided strong evidence that the benefits were due to the specific active ingredients of time management training, rather than non-specific effects or general mental health knowledge. It suggests that merely providing information about mental health problems and resources is not sufficient to produce the same level of improvement in symptoms and skills as a structured program teaching actionable coping strategies. This underscores the importance of moving beyond awareness-raising to the direct teaching of evidence-based psychological skills, even in low-intensity, digital formats.

The absence of mediation through problem-solving is likely explained by the nature of our active control group. Because the control condition received substantial psychoeducational materials about mental health, symptoms, and resources, it may have produced comparable improvements in perceived problem-solving skills. With no significant group differences in PSI change (i.e., the intervention failed to manipulate the mediator more than the control), statistical mediation cannot occur. This suggests that problem-solving is not a unique mechanism of this intervention and that the active control’s educational content may have inadvertently engaged similar cognitive processes.

It appeared that our ISSS did not significantly improve positive outcomes of mental health in general. It may reflect a crucial distinction in the mental health intervention landscape: the difference between reducing pathology and promoting flourishing. PST, a core component of our intervention, is fundamentally a deficit-based model (addressing helplessness, avoidance, and overwhelm) but is not designed to systematically build positive pillars, such as positive emotions, engagement, relationships, meaning of life, and accomplishment, in the flourishing model. Thus, it may not necessarily equip them to cultivate a garden of well-being on stable ground. Future research should investigate whether integrating PST with well-being promotion approaches (e.g., strengths-spotting, mindfulness, gratitude exercises) could create a more powerful, synergistic intervention that both alleviates suffering and elevates thriving.

A significant discrepancy between ITT and PP analyses for self-efficacy was identified. The significant time by group interaction was found for self-efficacy only among per-protocol participants (those who completed the intervention as intended), which aligns with Bandura’s social cognitive theory. Self-efficacy beliefs are theorized to develop through active mastery experiences, vicarious learning, and successful performance of targeted behaviors. Participants who dropped out or had low adherence likely did not receive sufficient exposure to these active ingredients, and their inclusion in the ITT analysis diluted the overall effect. This suggests that building self-efficacy may require a “full dose” of the intervention, including completion of core exercises and skill practice.

### Implications

As the first RCT evaluating digital mental health intervention for common mental health problems of school teachers in HK, ISSS addresses research and practical gaps in the literature and community service. First, by identifying time management as a significant mediator for long-term depressive symptoms, it moves beyond establishing if PST works and begins to elucidate how and for whom it works. This finding provides empirical support for the relational/problem-solving model of stress and suggests that time management is a core mechanism to be targeted in interventions for overwhelmed populations. Second, the transdiagnostic effectiveness of ISSS (impacting both depression and anxiety) underscores the value of targeting underlying transdiagnostic processes like adaptive coping, rather than disorder-specific symptoms.

For school administrators and mental health practitioners, the ISSS program presents a scalable, low-cost, and sustainable solution to address the mental health crisis among teachers. Its online, self-help format overcomes critical barriers such as stigma, cost, and lack of time. Specifically, our results suggest that training should explicitly integrate time management skills as a core component for sustaining well-being. The role of the e-coach, while low-intensity, was likely crucial for adherence and personalization; this guided self-help model should be considered a minimum standard for implementation. ISSS is not intended to replace traditional therapy for severe cases but can serve as a highly effective first step in a stepped-care model within schools, freeing specialist resources for teachers and students with more severe needs. It may alleviate the shortage of health care resources in HK.

### Limitations and future studies

Several limitations of this study should be noted. Since the ISSS intervention was conducted online, we did not have much information on the process, how engaged the participants were during the session, or how frequently they practiced the skills they learnt. While digital interventions can address potential barriers such as time commitment, location, and stigma compared with in-person treatment, the long-term effectiveness of such therapies may be limited [39]. Longer follow-up evaluations are warranted. In-depth interviews among the participants may help to better understand the process, benefits, acceptability, and barriers of the digital mental health implementation.

Moreover, only 15% of participants attended all five sessions, with most completing three sessions. This high attrition rate is consistent with previous studies of internet-based interventions [40, 41] and likely reflects the time constraints and workload pressures faced by HK secondary school teachers, for whom multi-session interventions may be burdensome. Despite low completion, significant ITT effects on depression and anxiety emerged, suggesting that even partial exposure may benefit mood. However, the PP effects on self-efficacy among completers suggest that deeper cognitive changes require fuller engagement, aligning with research indicating that longer therapy durations are needed for optimal outcomes [42–46]. An intervention that most participants do not complete has limited real-world impact, regardless of its efficacy for the minority who finish. From a pragmatic perspective, digital single-session interventions may be better aligned with teachers’ demanding schedules, potentially enhancing attendance motivation and completion rates.

While the use of an active control group (psychoeducation) is a methodological strength over a passive waitlist, it also presents a specific limitation. The observed effects of the ISSS intervention are relative to this specific control condition. It is possible that the psychoeducation materials themselves had a positive effect, potentially attenuating the measured differences between the groups. Therefore, our results demonstrate efficacy beyond a robust attention control but cannot speak to efficacy beyond a placebo effect or the specific impact of receiving mental health information.

Our recruitment strategy targeted teachers actively interested in receiving a mental health program. This self-selection bias means our sample likely comprised individuals with a higher pre-existing propensity to change and greater motivation to engage with self-help materials than the average teacher. Consequently, the high adherence rates and positive outcomes we observed may overestimate the engagement and effectiveness that could be expected in a universal, mandatory rollout to a more heterogeneous teacher population, which would include many individuals who are pre-contemplative about changing their coping strategies.

Finally, our sample consisted of a non-clinical population of teachers with predominantly mild to moderate symptoms (PHQ-9/GAD-7 ≥ 5). While this aligns with the preventive, selective intervention goals of the study, it limits the generalizability of our findings to teachers with more severe, clinical levels of depression or anxiety. The intervention’s effects might differ in a clinically diagnosed population, potentially showing smaller effects for severe cases requiring more intensive treatment or larger effects for those with subthreshold symptoms. The clinical significance of improvements in a predominantly mild-to-moderate sample warrants careful consideration. The Cronbach’s alpha for the PHQ at baseline was relatively low although acceptable. Its reliability improved to good levels at post-test and follow-up. Future studies using the PHQ-9 with HK teachers should examine test-retest reliability and consider whether a brief familiarization procedure might enhance baseline reliability.

## Supplementary Information

Below is the link to the electronic supplementary material.

Supplementary Material 1

## Data Availability

The data that support the findings of this study are available from the corresponding author, XY, upon reasonable request.
