# Supplementary material for: A randomized controlled trial of an Internet-based self-help skill strengthening (ISSS) intervention for secondary school teachers: a transdiagnostic intervention for common mental health problems
Source: BMC Psychiatry. 2026 Apr 17;26:429. doi: 10.1186/s12888-026-08063-4 (PMC13217987; doi:10.1186/s12888-026-08063-4)
Supplement: Supplementary file 1 — Supplementary Material 1 [file 12888_2026_8063_MOESM1_ESM.docx]

**Appendix 1**

Primary outcomes

**Depression**

The Patient Health Questionnaire (PHQ; Kroenke, Spitzer & Williams 2001) consists of nine questions designed to correspond to the nine diagnostic criteria for major depressive disorder covered in the Diagnostic and Statistical Manual of Mental Disorders (DSM–IV). Items are rated from 0 to 3 according to increased frequency of experiencing difficulties in each area covered. Scores are summed and can range from 0 to 27. Score 10 is often recommended as the cut-off score to detect major depressive disorder. Scores of 5, 10, 15, and 20 represent cut-off points for mild, moderate, moderately severe, and severe depression, respectively (Kroenke et al., 2001). The Chinese version has been validated (Wang et al., 2014).

**Anxiety**

Generalized Anxiety Disorder Scale (GAD) is a 7-item instrument developed to identify likely cases of generalized anxiety disorder in primary care patients and it is a widely-used screening tool of anxiety (Spitzer, Kroenke, Williams, & Löwe, 2006). Items are rated for the last two weeks, using a four-point rating scale from 0 to 3. A score of 10 or greater represents a cut point for identifying cases of generalized anxiety disorder, while cut points of 5, 10, 15, and 20 are interpreted as representing mild, moderate, moderately severe, and severe levels of anxiety (Spitzer et al., 2006). The Chinese version has been validated (Tong et al., 2016).

Secondary outcomes

**Perceived stress**

Symptoms of stress were measured with the Perceived Stress Questionnaire (PSQ) (Fliege et al., 2005; Levenstein et al.,1993). This questionnaire consists of 20 items measuring perceived stressful situations and stress reactions in four subdomains: worries, tension, joy, and demands. The respondent is asked to rate how often he or she experienced certain situations or reactions on a 4-point Likert-type scale (l=almost never, 2=sometimes, 3=often, 4=usually) during the past four weeks. Higher scores on the subdomains of worries, tension and demands denote a higher level of perceived stress, whereas a higher score for joy denotes a lower level of perceived stress. An average score of all item scores can be calculated. After a linear transformation the overall score ranges between 0-1. The reliability and validity of the Chinese version are adequate (Luo et al., 2018).

**Self-efficacy**

The 10-item General Self-Efficacy Scale (GSE) developed by Schwarzer and Jerusalem (2010) assesses a general sense of perceived self-efficacy, with the goal of predicting the ability to cope with daily problems and adapt after experiencing stressful life events. The participant is asked to evaluate statements on a 4-point Likert-type scale (l=not at all true, 2=hardly true, 3=moderately true, 4=completely true) (e.g., "I can typically handle whatever comes my way"). A higher score indicates higher self-efficacy. The item values can be summarized to a total score that ranges between 10-40. The Chinese version had good reliability and validity (Yang, Lau, & Lau, 2018).

**Job performance**

The measure of subjective job performance will be adopted from the Performance Maintenance Scale used in the study of Abraham and Hansson (1995). Participants are asked to indicate the extent to which they had been able to maintain 11 job-related abilities in their job in the past year, such as power or status at work, productivity, independence at work, efficiency, and career development. Items were rated on a 6-point scale, ranging from 1 (could not) to 6 (perfectly well). The Chinese version has been used in previous studies (Yeung & Fung, 2009).

**Well-being**

The 5-item World Health Organization Well-Being Index (WHO-5) is a short and generic global rating scale measuring subjective well-being. Because the WHO considers positive well-being to be another term for mental health, the WHO-5 only contains positively phrased items (Bech, 1999). The WHO-5 items are: (1) ‘I have felt cheerful and in good spirits', (2) ‘I have felt calm and relaxed', (3) ‘I have felt active and vigorous', (4) ‘I woke up feeling fresh and rested' and (5) ‘My daily life has been filled with things that interest me'. Participants are asked to rate how well each of the 5 statements applies to them when considering the last 14 days. Each of the 5 items is scored from 5 (all of the time) to 0 (none of the time). The Chinese version has been used in previous studies (Wu, 2014).

Mediators

**Problem solving**

The Problem Solving Inventory (PSI; Heppner & Petersen, 1982) is a 35-item self-report scale. It assesses an individual's awareness and evaluation of his or her problem-solving abilities or styles. Higher scores indicate greater problem solving awareness and ability. The Chinese PSI was shown to have significant associations with life stress, self-esteem, and depression scores in Chinese adolescents (Cheng & Lam, 1997).

**Time management behaviors**

The 33-item Time Management Behavior Scale (TMBS) developed by Macan et al. (1990) from a compilation of time management tips, ideas, and techniques will be used to assess subjects' use of time management behaviors. They cover topic areas including setting goals, prioritizing, organizing, and scheduling. The scale items assess the extent to which time management activities are used, not the individual's evaluation of the effectiveness or appropriateness of such behaviors. Participants rate each item using a 5-point Likert-type scale from seldom true (1) to very often true (5). Negatively worded items are reverse scored. Higher mean scores indicate more frequent use of time management.

**Reference**

Abraham, J. D., & Hansson, R. O. (1995). Successful aging at work: An applied study of selection, optimization, and compensation through impression management. Journal of Gerontology: Psychological Sciences, 50B, P94.

Bech, P. (1999). Health‐related quality of life measurements in the assessment of pain clinic results. Acta Anaesthesiologica Scandinavica, 43(9), 893-896.

Cheng, S. K., & Lam, D. J. (1997). Relationships among life stress, problem solving, selfesteem, and dysphoria in Hong Kong adolescents: Test of a model. Journal of Social and Clinical Psychology, 16, 343–355.

Fliege, H., Rose, M., Arck, P., Walter, O. B., Kocalevent, R. D., Weber, C., & Klapp, B. F. (2005). The Perceived Stress Questionnaire (PSQ) reconsidered: validation and reference values from different clinical and healthy adult samples. Psychosomatic medicine, 67(1), 78-88.

Heppner, P. P., & Petersen, C. H. (1982). The development and implications of a personal problem solving inventory. Journal of Counseling Psychology, 29, 66–75.

Kroenke, K., Spitzer, R. L., & Williams, J. B. (2001). The PHQ‐9: validity of a brief

depression severity measure. Journal of general internal medicine, 16(9), 606-613.

Levenstein, S., Prantera, C., Varvo, V., Scribano, M. L., Berto, E., Luzi, C., & Andreoli, A. (1993). Development of the Perceived Stress Questionnaire: a new tool for psychosomatic research. Journal of psychosomatic research, 37(1), 19-32.

Luo, Y., Gong, B., Meng, R., Cao, X., Tang, S., Fang, H., ... & Liu, B. (2018). Validation and application of the Chinese version of the Perceived Stress Questionnaire (C-PSQ) in nursing students. PeerJ, 6, e4503.

Macan, T. H. (1994). Time management: Test of a process model. Journal of applied

psychology, 79(3), 381.

Schwarzer, R., Jerusalem, M. 2010. The general self-efficacy scale (GSE). Anxiety, Stress, and Coping 12, 329-345.

Spitzer, R. L., Kroenke, K., Williams, J. B., & Löwe, B. (2006). A brief measure for assessing generalized anxiety disorder: the GAD-7. Archives of internal medicine, 166(10), 1092-1097.

Tong, X., An, D., McGonigal, A., Park, S. P., & Zhou, D. (2016). Validation of the Generalized Anxiety Disorder-7 (GAD-7) among Chinese people with epilepsy. Epilepsy research, 120, 31-36.

Wang, W., Bian, Q., Zhao, Y., Li, X., Wang, W., Du, J., ... & Zhao, M. (2014). Reliability and validity of the Chinese version of the Patient Health Questionnaire (PHQ-9) in the general population. General hospital psychiatry, 36(5), 539-544.

Wu, S. F. V. (2014). Rapid screening of psychological well-being of patients with chronic illness: reliability and validity test on WHO-5 and PHQ-9 scales. Depression research and treatment, 2014.

Yang, X., Lau, J. T., & Lau, M. C. (2018). Predictors of remission from probable depression among Hong Kong adolescents–A large-scale longitudinal study. Journal of affective disorders, 229, 491-497.

Yeung, D. Y., & Fung, H. H. (2009). Aging and work: How do SOC strategies contribute to job performance across adulthood?. Psychology and Aging, 24(4), 927.

Table Per-Protocol (PP) linear mixed models for primary and secondary outcome variables

|  | Control Mean (95%CI) | Intervention Mean (95%CI) | Group (F, p value) | Time (F, p value) | Group * time (F, p value) |
| --- | --- | --- | --- | --- | --- |
| PHQ | 7.50 (7.01, 7.99) | 6.16 (5.08, 7.23) | 4.97, .027 | 5.91, .003 | 2.35, .097 |
| GAD | 7.26 (6.69, 7.83) | 5.88 (4.64, 7.13) | 3.95, .048 | 7.37, <.001 | 3.51, .031 |
| GSE | 25.49 (24.79, 26.18) | 24.49 (22.97, 26.01) | 1.39, .240 | 2.10, .124 | 3.17, .043 |
| PSQ | 29.56 (28.88, 30.21) | 29.79 (29.12, 30.44) | .00, .300 | 0.97, .255 | 1.75, .176 |
| JP | 66.64 (64.65, 68.63) | 64.95 (60.70, 69.21) | .50, .480 | .71, .493 | .14, .869 |
| WHO-5 | 16.95 (16.34, 17.55) | 16.49 (15.19, 17.78) | .40, .526 | 9.18, <.001 | .44, .642 |
| PSI | 97.92 (96.42, 99.42) | 96.70 (93.53, 99.86) | .47, .492 | 8.52, <.001 | 1.04, .355 |
| TMBS | 115.29 (113.88, 116.71) | 123.22 (120.19, 126.25) | 21.77, <.001 | 1.82, .164 | .60, .548 |

*Note.* PHQ = depression; GAD = anxiety; GSE = self-efficacy; PSQ = perceived stress; JP = job performance; WHO-5 = well-being; PSI = problem-solving; TMBS = time management.
